# Implication of *OPRM1* A118G Polymorphism in Opioids Addicts in Pakistan: In vitro and In silico Analysis

**DOI:** 10.1007/s12031-018-1123-1

**Published:** 2018-07-22

**Authors:** Madiha Ahmed, Ihsan ul Haq, Muhammad Faisal, Durdana Waseem, Malik Mumtaz Taqi

**Affiliations:** 1 Department of Pharmacy, Quaid-i-Azam University, Islamabad, Pakistan; 2 Faculty of Health Studies, University of Bradford, Richmond Rd, Bradford, UK; 3 Bradford Institute for Health Research, Bradford Teaching Hospitals NHS Foundation Trust, Bradford, UK; 4 Division of Mental Health and Addiction, NORMENT, University of Oslo, Oslo, Norway

**Keywords:** μ-Opioid receptor, A118G polymorphism, Single nucleotide polymorphism (SNP), N40D

## Abstract

Single nucleotide polymorphism in *OPRM1* gene is associated with hedonic and reinforcing consequences of opioids. Risk and protective alleles may vary in different populations. One hundred healthy controls and 100 opioids (predominantly heroin) addicts from Pakistani origin were genotyped for A118G (N40D) polymorphism in *OPRM1*. Structural and functional impact of the polymorphism on encoded protein was predicted by in silico analysis. Results show significant association between homozygous GG genotype and opioid addiction in Pakistani population (*p* value = 0.016). In silico analysis by SIFT (TI = 0.61), PolyPhen (PISC = 0.227), PANTHER (subPSEC = −1.7171), and SNP effect predicted this SNP benign for encoded protein. Superimposing wild-type and mutated proteins by MODELLER shows no change (RMSD = 0.1) in extracellular ligand binding domain of μ-opioid receptor. However, Haploreg and RegulomeDB predicted *OPRM1* gene repression by chromatin condensation and increased binding affinity of RXRA transcription factor that may reduce protein translation and hence the number of available receptors to bind with drugs, which may trigger underlying mechanisms for opioids addiction. Thus, this study outlines causal relationship between opioids addiction and genetic predisposition in Pakistani population.

## Introduction

Drug addiction, characterized by repetitive and compulsive use of drugs to attain euphoria (Deroche-Gamonet et al. 2004), predisposes the addict to tolerance and elevate the risk of withdrawal symptoms upon reducing the intake of drug (Volkow and Li 2004). Persistent drug abuse triggers neurological changes leading to psychological and physical dependence, craving, and relapse (Camí and Farré 2003). Deregulation of endogenous opioid and dopamine systems mediate the hedonic and reinforcing consequences of addictive drugs (Camí and Farré 2003). It burdens an individual with the high costs associated with medical treatment, injuries, drug-related complications, crimes, incarceration, and time lost from work and social welfare programs.

Multiple factors including pharmacological and physicochemical properties of drugs, psychiatric discords, risk-seeking and novelty-seeking traits, and stressed life and dominantly genetic makeup may provoke an individual to abuse drugs (Crabbe 2002; FARRÉ and CAMÍ 1991; Helmus et al. 2001). A study evidenced higher incidence of alcohol addiction in individuals born to alcohol addict parents despite being adopted and raised by non-addict parents (Schuckit and Smith 2001). Genetic polymorphism in endogenous opioid system is associated with drug abuse (Kosten et al. 1986). Single nucleotide polymorphism (SNP) in *OPRM1* gene encoding μ-opioid receptor is significantly associated with opioid dependence and heterogeneous response to various ligands including β-endorphin, enkephalins, morphine, heroin, methadone, cocaine, and alcohol (Nestler 2001; Uhl et al. 1999). A missense SNP rs1799971 (A118G) located in exon 1 (Asn40Asp) has been extensively studied for its association with substance dependence in Caucasian (Bergen et al. 1997), European American, African American (Crowley et al. 2003), German (Mistry et al. 2014), and Japanese (Gelernter et al. 1999) populations.

A118G polymorphism removes a highly conserved N-glycosylation site in protein’s extracellular domain (Bergen et al. 1997) that may hamper pain perception in chronic diseases (Fillingim et al. 2005; Janicki et al. 2006), reduce response towards analgesic drugs (Oertel et al. 2009; Oertel et al. 2006), and tend to increase administration of opioids (Chou et al. 2006; Sia et al. 2008).Another missense SNP C17T in exon 1 (Ala6Val) (Zhang et al. 2007) was significantly linked with heroin addiction (Rommelspacher et al. 2001). Several rare variants such as Ser147Cys in exon 2 and Ile292Val in exon 3 of *OPRM1* gene have also been reported but their functional significance is not elucidated (Bergen et al. 1997). Moreover, *OPRM1* SNPs in intron 1 are associated with euphoric response to heroin in Chinese population (Zhang et al. 2007) and cocaine and opioid drugs’ dependence in European Americans (Zhang et al. 2006). Another study documented combined association of SNP A118G and SNP C1031G in intron 2 of *OPRM1* gene with heroin addiction (Szeto et al. 2001).

In Pakistan, drug abusers have raised from 4.1 million to 6.45 million. A recent report documented opioid abuse in 5.8% population aged 15–64 years in past 12 months (Organization 1992). It is imperative to identify genetic predisposition to opioid addiction in Pakistani population. The present study determines the association of drug addiction with SNP A118G in *OPRM1* gene in Pakistan. It also predicts implications of single nucleotide polymorphism on encoded protein structure and function, allele-specific transcription factor interactions, and chromatin structure to elucidate molecular mechanism underlying A118G association with drug addiction. The aim of the current study is to identify genetic predisposition to opioids addiction in Pakistani population that may enable us to take pre-emptive and preventive measure to reduce prevalence of opioids addiction in genetically vulnerable individuals. Moreover, it may also enable us to retrieve novel molecular and genetic targets to design safe and effective treatment for opioids addicts.

## Material and Methods

Blood samples from 100 healthy individuals (mean age 33.58 years) without history of psychotic disorder, drug abuse, and dependence and 100 opioid addicts (predominantly heroin; mean age 34.35 years) undergoing detoxification therapy at rehabilitation centers of Faisalabad and Lahore, Pakistan, were collected with the consent of test subjects and according to human ethical protocols provided by Declaration of Helsinki (World Medical Association 2001). Drug addicts were selected based on criteria for drug dependence described in diagnostic and statistical manual of mental disorders (American Psychiatric Association 2013). Structured questionnaires were used to gather demographic data, medical history, family history, and socioeconomic status of the individuals.

### Collection of Blood Samples

Blood samples were collected in EDTA impregnated vacutainers (3 ml, 13 × 75 mm, Ayset, Turkey) and stored at 4 °C until further analysis.

### DNA Extraction and Amplification

DNA was extracted by phenol chloroform method (Bell et al. 1981). Briefly, 750 μl blood was mixed with an equal volume of solution A [0.32 M sucrose, 10 mM Tris (pH 7.5), 5 mM MgCl_2_, 1% Triton X-100], incubated (25 °C, 30–40 min), and centrifuged (13,000 rpm, 1 min) to separate cell pellets. The cell pellets were re-suspended in 400 μl of solution A, centrifuged and again re-suspended in 400 μl of solution B [10 mM Tris (pH 7.5), 400 mM NaCl and 2 mM EDTA (pH 8)] along with 12 μl 2%SDS and 5 μl proteinase-K solutions. After incubation (37 °C, overnight) and centrifugation (13,000 rpm, 10 min), the aqueous phase was mixed with an equal volume of solution D [chloroform: isoamyl] and centrifuged again. Upper aqueous phase was mixed with an equal volume of chilled isopropanol and 55 μl 3 M sodium acetate solution (pH 6). Precipitated DNA was washed with 200 μl of 70% ethanol and dissolved in 200 μl of TE buffer [10 mM Tris (pH 7.5), 1 mM EDTA (pH 8)].

*Amplification of desired segment of OPRM1* gene was carried by previously reported PCR primers. Forward primer *5′-* CGGTTCCTGGGTCAACTTGTCCCACTTAGATCGC-3′ and reverse primer 5′-AGCCTTGGGAGTTAGGTGTCTC-3′ (Ginosar et al. 2009). The primers (Integrated DNA Technology, USA) were sized between 22 and 24 bases with a Tm of 69–71 °C and a GC content of 40–60%. DNA templates were added to a reaction mixture containing 1.25 μl of each primer (5 pmoles), 2.5 μl buffer (Bio Basic Inc., Canada), 2 μl MgSO_4_ (Bio Basic Inc., Canada), 0.3 mM dNTPs, and 0.04 units/μl of taq polymerase (Bio Basic Inc., Canada). The following PCR profile was used: denaturation for 5 min at 94 °C, 35 cycles for 1 min at 94 °C, 1 min at primer-specific annealing temperature (58 °C), and 2 min at 72 °C followed by final incubation at 72 °C for 4 min. Once amplified, the fragments were purified with GeneJET™ PCR Purification Kit (Fermentas, USA) and confirmed by 1% agarose gel electrophoresis using 50 bp ladder DNA molecular weight marker (Fermentas, Lithuania) (Ginosar et al. 2009).

### SNP Genotyping

SNP genotyping was done by restriction fragment length polymorphism (RFLP) and confirmed by sequencing. Purified DNA samples from control and experimental groups were digested with restriction enzyme Bsh 12361 (BstU1, #ER0921, 10 units/μl, Fermentas, Lithuania) and analyzed by Agilent Bioanalyzer 2100 (Agilent, USA) according to manufacturer’s instructions. Twenty-five samples from each group were randomly selected and sent for sequencing to Eurofins MWG Operon (Huntsville, AL). Sequencing was performed by dideoxy chain termination method (Sanger et al. 1977) using ABI 3730XL sequencer.

### Statistical Analysis

Genotype and SNP association with opioid (predominantly heroin) addiction was analyzed by chi square test supplemented by power analysis and determination of odd ratios (OD). Statistical analysis was done using statistical software R version 3.0.1.

### In silico Analysis of SNPs

In silico analysis of *OPRM1* SNP rs1799971 was done to predict its impact on encoded protein’s structure and function. SIFT (http://sift.bii.a-star.edu.sg/) predicts the phenotypic effect of mutation on protein structure, based on sequence homology and physical properties of amino acids (Ng and Henikoff 2003) and calculates tolerance index (TI) that is classified as intolerant (0.00–0.05), potentially intolerant (0.051–0.10), and tolerant (0.201–1.00). The higher the TI, the lesser the functional impact of mutation. Query was submitted as SNP Id with default settings (3.00 median conservation score, remove sequences > 90% identical to query sequence) and selecting SWISS-PROT and TrEMBL databases. Risk associated with amino acid substitution (AAS) was predicted by PolyPhen (Ramensky 2002). PolyPhen (http://coot.embl.de/PolyPhen/) predicts effect of AAS based on evolutionary conservations, physiochemical differences, and substitution vicinity to structural features of protein. Query was submitted as protein sequence (FASTA) along with substituted amino acids and their position.

SNPeffect (http://snpeffect.switchlab.org/) (Reumers et al. 2006) annotate the variant by algorithms like TANGO (predicts aggregation regions), WALTZ (for amylogenic region prediction), and FoldX (analyzes effect on structure stability) [34]. PANTHER (http://www.pantherdb.org/tools/csnpScoreForm.jsp) (Reumers et al. 2006) uses hidden Markov model (HMM)-based statistical methods to predict deleterious variants by calculating substitution position-specific evolutionary conservation (subPSEC) score ranging between 0 (neutral) and − 10 (most likely to be deleterious). P_deleterious_ determined the probability of given variant to cause a deleterious effect (Mi et al. 2013). The higher the value of P_deleterious_ score, the severe the impact of a variant on protein function. Protein sequence was used as input for SNP prediction.

To visualize the impact of mutation, A118G SNP was mapped on 3D structure of *OPRM1*. Since, the crystal structure of human *OPRM1* is not determined; therefore, we used 3D structure of *Mus musculus OPRM1* for homology-based prediction. The protein template (PDB ID: 4DKL) was selected from basic local alignment search tool (BLAST) with highest sequence identity and smallest distance on the phylogenetic tree. MODELLER (Martí-Renom et al. 2000) was used for prediction of 3D structure of *OPRM1*. RMSD between the mutant and wild-type protein structure was calculated to check the effect of mutation on stability of protein structure.

In addition, we used RegulomeDB (Boyle et al. 2012) and Haploreg V2 (Ward and Kellis 2012) to determine the effect of *OPRM1* SNP on chromatin structure and allele-specific transcription factor binding. HaploregV2 (http://www.broadinstitute.org/mammals/haploreg/haploreg.php) discovers variants present on haplotype blocks and explores their regulatory nature and linkage with disease associated loci (Ward and Kellis 2012). We used European and American population groups to retrieve the SNPs present in LD with our lead SNP. RegulomeDB (http://regulomedb.org) utilized CHIP-seq data and chromatin state information across many cell types as well as expression quantitative trait loci (eQTL) information for functional annotation of variants (Boyle et al. 2012). This data was retrieved by using SNP Ids. RegulomeDB scores the variants based on predicted potential effects caused by the variant residing in a functionally important region of the genome. The lower the score, the higher is the effects on protein binding and expression of target gene (Boyle et al. 2012).

## Results

### Association of *OPRM1* SNP with Opioid Addiction

Genotyping of *OPRM1* SNP rs1799971 by RFLP and sequencing methods show significant association of A118G variant with addiction in Pakistani population. Among 35 females and 165 males, homozygous genotype A/A was found with highest frequency in both control and experimental groups while heterozygous genotype A/G was more frequent in control group (Table 1). Moreover, frequency of mutated G-allele was higher in addicts as compared to control group (Table 1) indicating a higher risk for the opioids addiction.

**Table 1:** Genotype and allele frequencies in control group and drug addicts

Groups	Genotype			Allele frequency	
	A/A	A/G	G/G	A	G
Control (*N* = 100)	79	13	8	171 (0.86)	29 (0.14)
	79%	13%	8%	85.5%	14.5%
Addicts (*N* = 100)	71	07	22	149 (0.74)	51 (0.26)
	71%	7%	22%	74.5%	25.5%

**Fig. 1:**
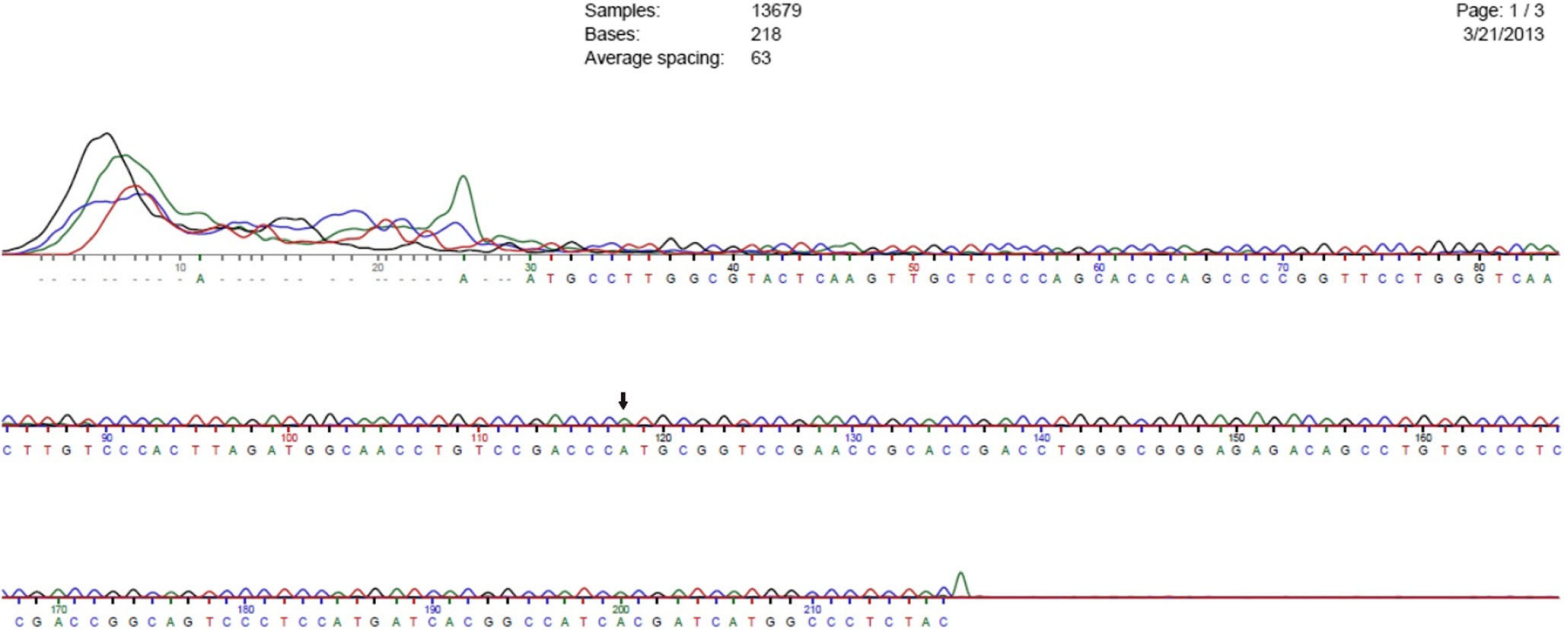
Sequence chromatogram for sample C9. Sequencing of OPRM1 gene in the DNA samples from control group was performed using ABI 3730XL sequencer. Arrow mark represents wild-type nucleotide A at position 118 in exon 1 of OPRM1 gene

**Fig. 2:**
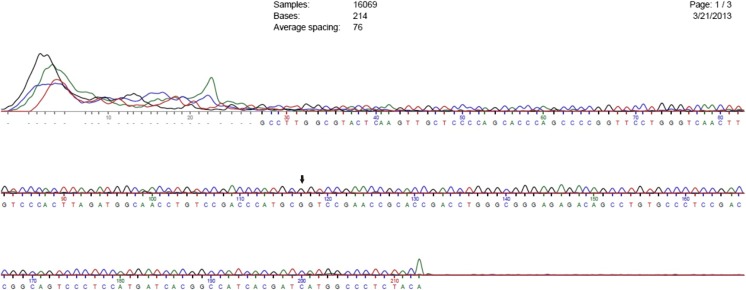
Sequence chromatogram for sample D10. Sequencing of OPRM1 gene in the DNA samples from opioid addicts was performed using ABI 3730XL sequencer. Arrow mark represents mutated nucleotide G at position 118 in exon 1 of OPRM1 gene

Wild-type allele = A, mutated allele = G, heterozygous genotype = A/G, homozygous genotype = A/A, G/G,

The genotypic and allelic frequencies of A118G variant (Figs. 1 and 2) depict significant association of opioid addiction with GG genotype in addicts (Table 2). Genotype distribution in control group and drug dependents was not in Hardy-Weinberg equilibrium (*p* < 0.05). The +118AA genotype was taken as reference due to its higher frequency in control group. Power analysis of these results is significant for the strength of performed tests (Table 2).

**Table 2:** Distribution of A118G polymorphism in *OPRM1* gene between control group and drug addicts

Groups	Genotypic frequency				Allelic frequency		HW *χ*^2^ value (*p*)
	AA	A/G	G/G	AG + GG	A	G	
Control	79	13	8	21	171 (0.86)	29 (0.14)	22.62 (< 0.001)
Addicts	71	07	22	29	149 (0.74)	51 (0.26)	67.54 (< 0.001)
Odds ratio	1	0.60	3.06	1.54	0.49	2.02	–
95% CI	–	0.23–1.59	1.28–7.31	0.81–2.95	0.30–0.82	1.21–3.34	
*χ* ^2^	–	0.64	5.77	1.31	–	–	–
*P*	–	0.419	0.016	0.253			
RR	–	–	–	–	0.73	1.37	–
*P*	0.7175			–	0.8074		

*HW χ2* Hardy-Weinberg chi square, *CI* confidence interval, *p p* value, *RR* relative risk

### Functional Annotation of rs1799971

In silico analysis of SNP rs1799971 by SIFT and PolyPhen characterized mutation N40D as tolerant and benign (sensitivity, 0.91, specificity = 0.88) respectively to protein structure. Similar effect was predicted by PANTHER. Furthermore, physicochemical properties of mutated protein analyzed by SNPeffect predicted change neither in protein aggregation nor amyloid propensity (Table 3). FoldX did not provide output due to unavailability of *OPRM1* protein 3D structure.

**Table 3:** Phenotypic and functional annotation of A118G polymorphism

dbSNP ID	rs1799971		PANTHER		SNPeffect	
Ch:Position (hg19)	6: 154360797		subPSEC	− 1.7171	d_TANGO_	2.28
Function	Missense		P _deleterious_	0.21706	Prediction	No effect on the aggregation tendency
Allele	A/G		P _substituted_	0.05496	d_WALTZ_	− 0.47
AAS	N40D		NIC	1.1	Prediction	No effect on the amyloid propensity
LD (*r*^2^)	1		SIFT			
RegulomeDB score	4		TI		0.61	
eQTL	**---**		Prediction		Tolerated	
Regulatory motifs altered	LOD score		PolyPhen			
	Ref	Alt	PSIC		0.227	
Hic1	11.1	1.9	Prediction		Benign	
RXRA	1	13				

*AAS* amino acid substitution, *NIC* number of independent counts, *PSIC* position-specific independent counts, *subPSEC* substitution position-specific evolutionary conservation, *TI* tolerance index, *Ref* reference allele, *Alt* alternative allele; (---) indicates data no found

**Fig. 3:**
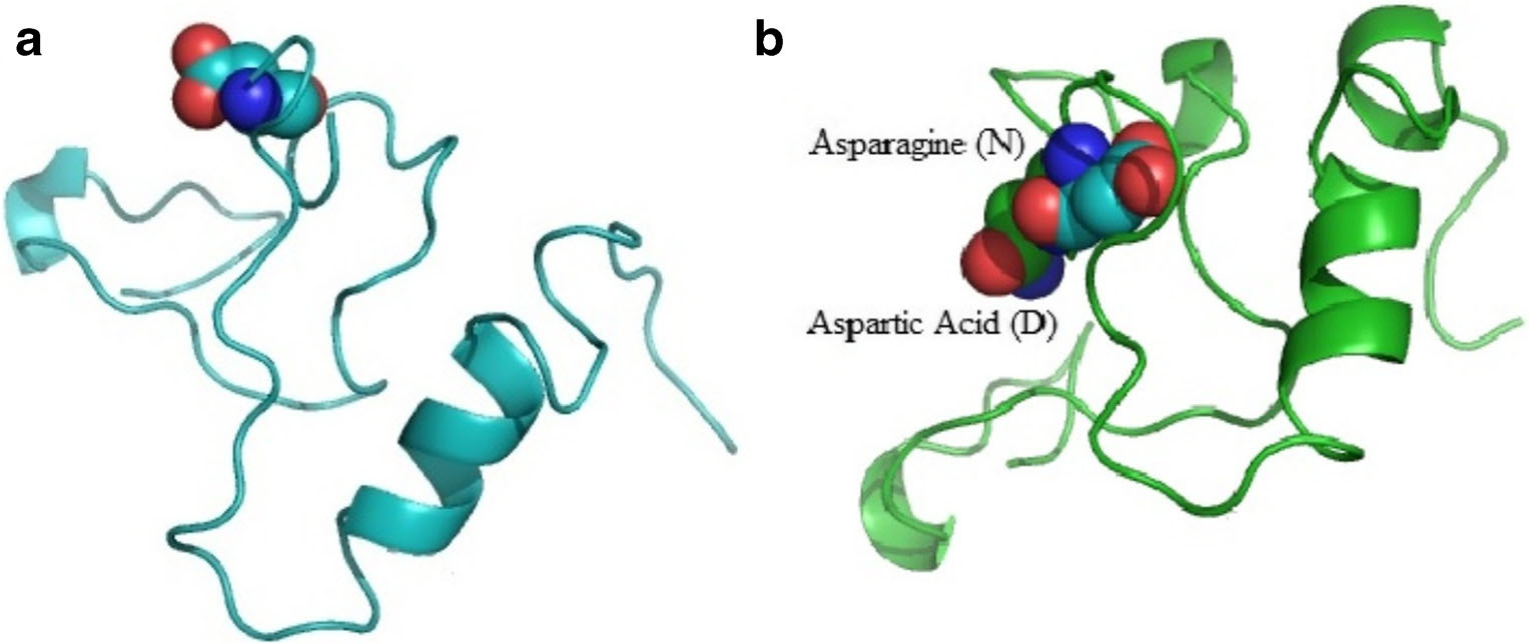
3D structure of extracellular domain of OPRM1 gene. a Mapped mutation D40, b Superimposed structures of wild-type N40 (cynic) and mutated D40 (green) protein

3D structure of *OPRM1* protein extracellular domain was predicted by MODELLER using energy score of models and ERRAT score (Fig. 3a). No change was observed in the structural domains of protein (RMSD = 0.1) after superimposing the native and mutated protein models and inducing mutation at corresponding position (N40D) by PyMOL viewer (Fig. 3b).

Furthermore, Regulome DB score 4 shows minimal binding evidence (Table 3) while the chromatin is condensed in different cell types (Table 4) indicating reduced expression of *OPRM1* gene in drug addicts by A118G polymorphism. Haploreg V2 shows no other SNP in LD with rs1799971. Moreover, A to G polymorphism creates canonical motif “AGARGGC**G**” for retinoid X receptor alpha (RXRA), whereas affinity of hypermethylated in cancer 1 (Hic 1) transcription factor is reduced due to loss of its motif “NBBRTGCC**A**MCCNRHH” (Table 3). These modifications repress transcription of *OPRM1* gene.

**Table 4:** Effect of A118G polymorphism on chromatin structure

Method	Location (hg19)	Histone mark	Cell type	Chromatin
ChIP-seq	chr6:153264243..154539751	H3k09me3	Nhdfad	Condense
ChIP-seq	chr6:154360700..154360850	H3k27me3	Hre	Condense
ChIP-seq	chr6:154360660..154360810	H3k27me3	Monocd14ro1746	Condense
ChIP-seq	chr6:154096836..154450021	H3k09me3	Nha	Condense
ChIP-seq	chr6:154110822..154537276	H3k27me3	Gm12878	Condense
ChIP-seq	chr6:154279593..154423747	H3k9me3	K562	Condense

## Discussion

Drug addiction is a developmental and neurological disorder influenced by genetic, behavioral, and environmental factors (Deroche-Gamonet et al. 2004; Volkow and Li 2004; FARRÉ and CAMÍ 1991; Crabbe 2002; Camí and Farré 2003; Helmus et al. 2001). In present study, we found significant association of SNP rs1799971 (A118G) with opioid addiction in Pakistan. We also predicted that A118G polymorphism does not change encoded protein structure; rather, it may repress *OPRM1* transcription and number of available receptors to bind with drugs by chromatin condensation and allele-specific transcription factor binding.

Results of this study reveal that frequency of mutated G-allele in our study is higher than Caucasian, European American, African American, German, and Swedish populations (Crowley et al. 2003; Barr et al. 2001; Gelernter et al. 1999; Bart et al. 2004; Bart et al. 2005; Hernandez-Avila et al. 2003), while it is lower as compared to Japanese, Chinese, Malay, Korean, Taiwanese, and Indian Asian populations (Kim et al. 2004; Li et al. 2000; Loh el et al. 2004; Nagaya et al. 2012; Nishizawa et al. 2006; Tan et al. 2003). This SNP is also associated with reduced effectiveness of morphine as an analgesic (Janicki et al. 2006). Nevertheless, a study has also reported no association of A118G polymorphism with drug addiction (Coller et al. 2009). This indicates that there is variability in implications of *OPRM1* gene polymorphism on drug addiction among different populations. Several factors contribute to false positive results in studies; for example, utilization of subjects belonging to mixed populations with craving for various substances (Bond et al. 1998) and small samples size (Nagaya et al. 2012). However, strong power analysis in our study supports significance of relationship between A118G polymorphism and opioids addiction. SNPs also affect pharmacological response of drugs. Studies have documented better response of opioid antagonist naloxone with good hypothalamic-pituitary-adrenal (HPA) axis activity in individual carrying 118G-allele (Hernandez-Avila et al. 2003; Wand et al. 2002).

We adopted most commonly used computational tools (Hou and Zhao 2013) for predicting the impact of A118G polymorphism in opioids addicts individuals. A recent review discussed the merits and demerits of these computational tools in detail (Nishizaki and Boyle 2017).

Computational analysis predicted A118G mutation non-damaging to extracellular domain of encoded protein indicating that it may not affect ligand binding affinity for μ-opioid receptor. Our results are in accordance with a previous study (Beyer et al. 2004), which reported unaltered binding affinity of morphine, morphine-6-glucuronide, and β-endorphin for both wild-type and mutated (N40D) μ-opioid receptors and demonstrated reduced expression of mutated receptors. This indicates that A118G mutation in *OPRM1* gene declined the expression of μ-opioid receptor in drug addicts decreasing the number of receptors available to interact with drugs. This is proved in our study by in silico analysis that A118G polymorphism causes chromatin condensation ultimately reducing transcription of *OPRM1* gene. Moreover, the absence of a ligand RXRA forms heterodimer with retinoic acid receptor alpha **(**RARA) associating with transcription co-repressor complex that causes chromatin condensation and transcriptional repression (Kastner et al. 1995; Mangelsdorf and Evans 1995). It is previously reported that RXRA-RARA complex repress transcription of multidrug resistance-associated protein (MRP3) (Chen et al. 2007). This shows that in the presence of opioids, RXR-RAR complex will activate μ-opioid receptor to attain hedonic effects while it modulate drug-seeking behavior and compulsive drug administration by repressing *OPRM1* gene in the absence of ligand.

Our study demonstrates significant association of opioid addiction with A118G polymorphism in Pakistani population. This SNP repress *OPRM1* transcription by chromatin condensation and altering regulatory motifs in an allele-specific manner. This corresponds to reduced μ-opioid receptor expression decreasing the number of receptors available to interact with drugs in opioid addicts. This study provides significant causal relationship between opioid addiction and genetic predisposition.

## Abbreviations

SNP: Single nucleotide polymorphism
Tm: Melting temperature
GC content: Guanine cytosine content
HPA: Hypothalamus pituitary axis
